# Impact of radiotherapy on the morphological and compositional structure of intra-radicular dentin

**DOI:** 10.1590/0103-6440202305101

**Published:** 2023-03-06

**Authors:** Georgia Ribeiro Martini, Eduardo A. Bortoluzzi, Mariana C. Minamisako, Natalia C. Trentin Bordignon, Paulo M. Rodrigues, Rogério Gondak

**Affiliations:** 1 Programa de Pós-Graduação em Odontologia, Universidade Federal de Santa Catarina (UFSC), Florianópolis, Santa Catarina, Brasil; 2 Centro de Pesquisas Oncológicas(CEPON), Florianópolis, Santa Catarina, Brasil; 3 Departamento de Patologia, Universidade Federal de Santa Catarina(UFSC), Florianópolis, Santa Catarina, Brasil

**Keywords:** intra-radicular dentin, radiotherapy, scanning electron microscopy

## Abstract

Considering the side effects in the oral cavity and dental structures of radiotherapy (RDT) for head and neck cancer, this study aimed to evaluate the effects of RDT on the root dentin concerning the obliteration of dentinal tubules, the inorganic composition of intra-radicular dentin, and the integrity of collagen fibers. Thirty human canines were selected from a biobank and randomly divided into two groups (n=15). The samples were sectioned buccolingually, and a hemisection was used for structural analysis by scanning electron microscopy (SEM) and energy-dispersive X-ray spectrometer (EDS). Low-vacuum SEM images were obtained at 2000-x magnification to observe the obliteration of the dentinal tubules. Moreover, compositional evaluation was performed using EDS. After RDT, the SEM and EDS analyses were repeated using the same methodology. RDT was applied fractionally at 2 Gy per day, 5 days per week, for 7 weeks, resulting in a total dose of 70 Gy. The collagen integrity of the irradiated and non-irradiated samples was analyzed using Masson’s trichrome and picrosirius red staining polarization microscopy. Samples subjected to RDT exhibited dentinal tubule obliteration (p < 0.001); low integrity of type I and III collagen fibers (p < 0.05); compositional reduction of calcium (p = 0.012), phosphorus (p = 0.001), and magnesium (p < 0.001); an increased Ca/P ratio (p < 0.001). RDT affects the structure of dentinal tubules, the inorganic composition of intra-radicular dentin, and the collagen fiber integrity in the root dentin, which may interfere with the effectiveness and durability of dental procedures.

## Introduction

Head and neck cancers are a group of tumors that affect the oral cavity, lips, nasal cavity, pharynx, larynx, and salivary glands ^1^. Radiotherapy (RDT) is one of the main forms of treatment, which is indicated after surgery and/or concomitantly with chemotherapy ^1^.

RDT uses electromagnetic waves to destroy tumor cells through the breakage of DNA molecules, preventing duplication ^1^. Despite its benefits for locoregional disease control, RDT is not selective for tumor cells and affects adjacent areas such as the mucosa, bone tissue, salivary glands, and teeth ^2^.

Previous reports have shown that RDT affects the tooth structure by reducing microhardness ^3^ ^,^ ^4^, obliterating dentinal tubules ^5^, causing enamel cracks and fissures, and altering the chemical composition of dentin in both permanent ^6^ and deciduous teeth ^3^. These changes can compromise dental procedures, as the bond strength of some restorative materials and endodontic filling cement depends on the integrity of collagen fibers and the exposure of dentinal tubules ^7^.

Several studies have evaluated the organic and inorganic structural compositions of coronary dentin; however, few studies have analyzed the effect of RDT on intra-radicular dentin. Therefore, this study aimed to evaluate, in vitro, the obliteration of dentinal tubules, the inorganic composition of intra-radicular dentin, and the integrity of collagen fibers in root dentin before and after RDT. The null hypothesis of this study was that RDT would be unable to cause a deleterious effect on the structure of the root dentin.

## Materials and methods

### Sample selection

Thirty healthy human canines were obtained from the UNOESC Biobank (Joaçaba, Santa Catarina, Brazil). All samples had straight canals, fully formed roots, and no curvature or apical resorption. The Research Ethics Committee (CAAE 79772117.0.0000.0121) approved this study. The samples were randomly divided for scanning electron microscopy (SEM), energy-dispersive X-ray spectrometry (EDS) (n=15), and microscopic analyses (n=15).

Sample preparation

The teeth were sectioned buccolingually with the help of a double-sided diamond disc (KG Sorensen, São Paulo, Brazil) coupled at a low speed (Kavo, Joinville, Santa Catarina, Brazil) under constant cooling to obtain two slices per root. The samples were then washed in running water, and a hemisection (n=15) was used for structural analysis using low-vacuum SEM and EDS (TM3030, Hitachi, Hefei Anhui, China). For microscopic evaluation, the samples were identified and sectioned following the aforementioned protocol. Additionally, one hemisection was submitted to the RDT protocol while the other, being the control group, was stored in deionized water for the same amount of time as the RDT.

### Evaluation in SEM and EDS

SEM and EDS evaluations were performed at the Center for Research in Ceramic and Composite Materials (CERMAT, UFSC, Santa Catarina, Brazil). Samples were identified and manually marked on the dentin, in the cervical third of the interior of the root canal, with a device with a 1 mm diameter circular cutting tip (Disposable Dermatological Punch, Miltex, Japan) to evaluate the same area before and after RDT. To remove all moisture, the samples were dehydrated in a container containing silica and kept in an oven at 37°C for 72 hours.

The samples did not undergo any coating or additional preparation. With the aid of double-sided tape, the samples were fixed and positioned in a standardized way on stubs and taken for SEM evaluation, with the option of low-vacuum operation at 2000x magnification (TM3030, Tabletop Microscope, Tokyo, Japan) to examine the dentin structure inside the root canal.

**Figure 1:**
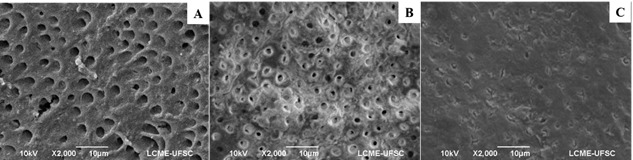
Classification of dentinal tubules by scanning electron microscopy (SEM). A, regular; B, partially obliterated; C, totally obliterated.

Dental images were digitally captured, and root dentin was evaluated using descriptive analysis by observing the obliteration of the dentinal tubules. The same orientation of the dentinal tubules was confirmed before the evaluation in all samples. Scores were attributed to the structure of the dentinal tubules: (0) regular, (1) partially obliterated, and (2) totally obliterated, as described by Gonçalves et al. (6) (Figure 1).

**Figure 2:**
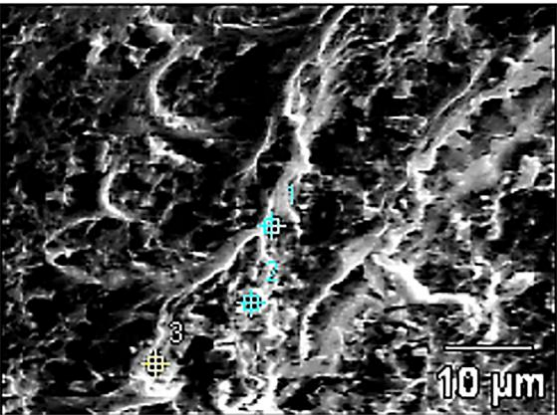
Mineral composition analysis by Energy-Dispersive X-ray Spectrometer (EDS) in 3 points of intra-radicular dentin.

The chemical composition of the dentin in the 15 samples was evaluated using EDS. The analysis was performed at three points on the dentin, both in the cervical third and inside the root canal (Figure 2). Following the first analysis, the samples were then subjected to RDT, and subsequently, SEM and EDS analyses were repeated.

### Microscopy evaluation of collagen integrity

**Figure 3:**
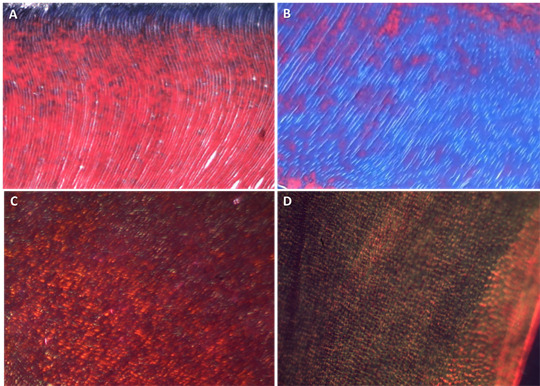
Evaluation of collagen fibers in the intra-radicular dentin of irradiated and non-irradiated teeth. Intense loss of integrity of collagen fibers of intra and peritubular dentin of an irradiated tooth (A) when compared to a non-irradiated tooth (B) (Masson's trichrome, 400x). Considerable loss of integrity of type I and III collagen fibers in the dentin of the irradiated tooth (C) when compared to the non-irradiated tooth (D) (Picrosirium red under polarization, 400x).

For microscopic evaluation of the collagen integrity of the root dentin, control and irradiated samples were decalcified with 5% ethylenediamine tetraacetic acid (EDTA) (pH 7.4) ^8^. The volume of the solution was at least 20 times greater than that of the sample. Decalcification occurred between 60 and 90 days. After decalcification, the samples were embedded in paraffin, sectioned at 3 µm, and placed on glass slides (Sigma-Aldrich, St. Louis, MO, USA). The slides were stained with Masson’s trichrome and picrosirius red under polarization. The analysis was performed using a polarizing microscope (Nikon Eclipse Ni polarizing microscope, Nikon Instruments, Tokyo, Japan) coupled with a digital image capture system (DS-Fi1c digital camera, Nikon, Tokyo, Japan) (Figure 3). Scores were assigned according to the integrity of collagen fibers in root dentin: unacceptable (-), low integrity (+), moderate integrity (++), and high integrity (+++) ^8^.

### RDT protocol

The teeth were placed on plastic support and immersed in deionized water, according to a previously described protocol ^9^. Computed tomography and specific software (Varian Eclipse, version 11.0.47) were used to plan the RDT protocol, which consisted of the application of 70 Gy divided into 2 Gy per day, 5 days a week, for 7 weeks. This approach was similar to the protocol used for the treatment of head and neck cancer in a previous study ^4^. RDT was performed at the Radiotherapy Department of Oncology Center Research (CEPON; Florianópolis, Santa Catarina, Brazil). The RDT equipment used was a Clinac 600 C/D, 6MV linear accelerator (Varian, Palo Alto, California, USA).

### Statistical analysis

The dentinal tubules and collagen fibers were statistically evaluated using the chi-square test. The EDS results were analyzed using the t-test for independent samples after confirming the normality of the data using the Shapiro-Wilk test. The statistical software SPSS (version 22.0) was used and the significance level was set at 5%.

## Results

The integrity of the dentinal tubules in the irradiated and non-irradiated samples was statistically different (p < 0.05). After RDT, 66.7% of the samples were classified as totally obliterated dentinal tubules, whereas 6.8% received the same classification as that before RDT. In the evaluation prior to RDT, the highest incidence was for dentinal tubules classified as regular (45.5%) and partially obliterated (47.7%) (Table 1).

**Table 1:** Evaluation of dentinal tubules before and after radiotherapy in dental samples.

	Dentinal tubules			
Group	Regular	Partially obliterated	Totally obliterated	*P*-value*
Before RDT	45.5%	47.7%	6.8%	< 0.05
After RDT	4.5%	28.8%	66.7%	< 0.05

RDT, Radiotherapy. *Chi-square test

In the analysis of collagen fibers, a considerable loss in the integrity of type I and II collagen fibers was observed in 62.5% of the irradiated samples, which significantly differed from what was observed in the non-irradiated group (p < 0.05) (Table 2).

**Table 2:** Evaluation of collagen fibers in the irradiated and non-irradiated groups.

Group	Low integrity	Moderate integrity	High integrity	*P*-value*
Control	0	22.2%	77.8%	< 0.05
RDT	62.5%	25%	12.5%	< 0.05

RDT, Radiotherapy. *Chi-square test

A compositional reduction in calcium (Ca) (p = 0.012), phosphorus (P) (p = 0.001), and magnesium (Mg) (p < 0.001) was observed after RDT, and the Ca/P ratio increased (p < 0.001) (Table 3).

**Table 3:** Compositional evaluation (mean and standard deviation) of dental samples before and after radiotherapy.

Elements	Before RDT	After RDT	*P*-value*
C	19.81 ± 10.96	27.88 ± 11.08	< 0.05
O	45.65 ± 6.03	44.36 ± 5.31	0.256
Mg	0.88 ± 0.21	0.51 ± 0.10	< 0.001
P	11.40 ± 3.06	9.19 ± 3.08	0.001
Ca	22.23 ± 8.26	18.10 ± 7.99	0.012
Ca/P	1.92 ± 0.28	2.06 ± 0.88	< 0.001

*RDT, Radiotherapy.* C, carbon; O, oxygen; Mg, magnesium; P, phosphorus; Ca, calcium. * T test for independent samples, significance level 5%.

## Discussion

The null hypothesis was rejected because RDT altered the dentinal tubular morphology, modified the inorganic composition of the intra-radicular dentin, and affected the integrity of the collagen fibers in the root dentin. A greater number of obliterated dentinal tubules was observed after RDT, demonstrating its deleterious effect on the tooth structure and corroborating the previous results by Velo et al. ^5^ and with other studies that evaluated the coronary dentin, both in primary ^3^ ^,^ ^10^ and permanent teeth ^6^.

Obliteration of the dentinal tubules has been associated with the deposition of residues resulting from cutting the dentin ^11^ degradation of the mineral structure ^5^, alteration in collagen fibers ^3^ ^,^ ^5^ ^,^ ^6^ ^,^ ^10^, or degradation of odontoblastic processes caused by the action of free radicals resulting from RDT ^10^ ^,^ ^12^. In our study, samples that already showed complete obliteration of the dentinal tubules in the initial evaluation were discarded, as this condition could be due to the cut of the sample. In addition, the loss of minerals from root dentin such as calcium and phosphorus resulting from RDT can cause the formation of cracks and obliteration of the dentinal tubules ^5^.

As for the alteration of collagen fibers, approximately 20% of the dentin is composed of organic matrix mainly composed of collagen, and RDT can influence the integrity of type IV collagen in the organic matrix, resulting in the fragility of this structure and favoring the fracture of the dental element ^13^. Another factor that can compromise the integrity of collagen fibers is the loss of hydration resulting from the action of free radicals by RDT ^6^ ^,^ ^14^.

The structure of the intra-radicular dentin is composed of dentinal tubules that extend from the pulp to the cement-dentin junction ^15^, which is composed of hypermineralized peritubular dentin with little organic matrix and intertubular dentin composed of type I collagen reinforced by apatite ^16^. Furthermore, the root dentin has a lower density of tubules, and a denser organic matrix than that the coronary dentin because it contains less peritubular and more intertubular dentin ^15^. Thus, the morphological changes in the organic and inorganic matrix resulting from RDT can cause the obliteration of the dentinal tubules.

In our study, most of the irradiated teeth showed low collagen integrity, whereas, in the control group, the collagen fibers remained intact in 77.8% of the cases, corroborating other studies ^6^ ^,^ ^17^. Campi et al ^17^ evaluated Fourier transform infrared spectroscopy alterations in collagen structure through the reduction of amide I, which is related to damage to the primary structure of collagen fibers ^18^.

The literature regarding the microscopic evaluation of the effects of RDT on collagen structure is scarce. However, histological studies are important because they allow a better understanding of this structure, and the Masson trichrome and picrosirius red stains under polarization allow the evaluation of collagen fibers ^19^. This technique enables the assessment of the degree of maturation and thickening of collagen fibers and allows the differentiation of type I and III fibers ^20^. Type I fibers are more prevalent in the organic part of the dentin, while type III fibers are less prevalent. In our study, RDT affected both types I and III fibers.

The effects of RDT on the obliteration of dentinal tubules and alteration in the structure of collagen fibers may be due to fiber fragmentation resulting from dehydration and the action of free radicals, leaving the tissue dehydrated and friable ^6^. Dentin has a composition of 10% water, and RDT interacts with water molecules by breaking their bonds, which generates free radicals that can alter and damage the organic matrix, thus affecting the structure of root dentin ^5^ ^,^ ^6^.

RDT is also associated with increased matrix metalloproteinase expression and activation in other organs ^21^. This enzyme is also responsible for the degradation of the organic matrix of dentin, contributing to collagen degradation and demineralization in the presence of dental caries ^22^. An increase in metalloproteinase 20 activity has also been reported in irradiated teeth ^23^. However, no consensus has yet been reached. Gomes et al. ^24^ did not observe an increase in metalloproteinase 2 and 9 levels in teeth subjected to head and neck radiotherapy.

Alterations in the organic matrix of the intra-radicular dentin can negatively affect the microhardness of the dentin structure ^6^, tooth resistance to fractures ^4^, and adhesion of dental materials and endodontic cements used in the filling of root canals ^7^. Endodontic protocols to reduce these deleterious effects are being researched ^7^.

RDT can also influence the inorganic components of dentin, as observed in our study with the reduction in Ca, P, and Mg after RDT. Additionally, Ca reduction has also been observed in root dentin ^5^ and enamel ^25^. The reduction in P also conforms with other studies ^3^ ^,^ ^17^, even though they have evaluated it in the form of phosphate. Moreover, the methodology used in our study, with EDS, evaluated the percentage of chemical elements in the sample, and not in another molecular format. The reduction in Ca and P in intra-radicular dentin suggests a less mineralized structure, which would facilitate the use of instruments during endodontic procedures and could affect the adhesion of some endodontic sealers ^26^. In contrast, Velo et al. ^5^ observed an increase in the percentage of P in irradiated teeth.

The Ca/P ratio determines the average mineralization of hydroxyapatite. In our study, an increase in this value was observed after RDT, which may explain the obliteration of dentinal tubules from an increase in peritubular dentin, which is more mineralized. However, this increase in hydroxyapatite in the intra-radicular dentin does not necessarily mean an increase in the mechanical strength of the analyzed region, because ions are released from the interaction between water and radiation, inducing the formation of a type of hydroxyapatite that is more susceptible to degradation ^27^. Velo et al. ^5^ who reported reduction in the Ca/P ratio after RDT of root dentin, resulting in less mineralized tissue, observed the opposite. Miranda et al. ^25^ found the same condition in the evaluation of enamel. This divergence between studies may be due to the different areas evaluated, since our study evaluated the inorganic composition of the intra-radicular dentin, while the other studies evaluated the buccal aspect of the root dentin ^5^ and enamel ^25^.

In this study, a reduction in Mg was observed. Mg is associated with tooth biomineralization ^28^ and directly affects the processes of crystallization and formation of the inorganic component of the tooth ^28^, which could contribute to a reduction in mineralization. Structural changes can make the dental substrate more friable and dehydrated ^6^, thereby compromising the mechanical strength of the tooth. Furthermore, the indirect effect of RDT caused by hyposalivation, which can intensify damage to dental structures, must be considered ^5^. In this sense, the radiation protocol for in vitro studies should keep the teeth hydrated, focusing only on the direct effect of RDT on the dental structure, avoiding dehydration. In our study, deionized water was used during RDT because depending on the storage medium, new ions and chemical components could have been incorporated into the samples, influencing the results.

The radiation dose used was based on RDT treatment for head and neck cancer, which usually involves a total dose of 70 Gy in the tumor region ^2^. An in vitro approach was chosen to use each sample as its control, comparing the outcomes before and after RDT, as different teeth can exhibit microstructural and compositional variations in dentin ^29^.

Studies on the deleterious effect of RDT on tooth structure are controversial ^3^ ^-^ ^5^ ^,^ ^21^ ^,^ ^24^. The difference in these results could be associated with the lack of a standardized methodology to assess the effect of radiation on dental structures. Some studies used bovine teeth ^4^, different radiation doses ^3^ ^-^ ^7^ ^,^ ^9^, and different RDT application protocols ^5^ ^,^ ^6^ ^,^ ^9^ ^).^

Due to the lack of a standardized methodology to assess the effects of RDT on tooth structure, it becomes difficult to compare our results with those of other studies, which is a limitation of the present study. Moreover, different doses of RDT can generate different effects, and in our study, we evaluated the maximum dose treatment prescribed for a patient with head and neck cancer.

RDT can cause deleterious effects on the intra-radicular dentin, such as obliteration of dentinal tubules, alteration in inorganic components, and harmful effects on the collagen fibers of the root dentin. This can interfere with the effectiveness of dental procedures such as root canal filling in endodontic treatments, and these changes must be considered when choosing endodontics or restorative materials. However, further studies are needed to better understand the deleterious effects of RDT, and protocols must be defined to minimize clinical consequences.
